# Cost-Effective Production of L-DOPA by Tyrosinase-Immobilized Polyhydroxyalkanoate Nanogranules in Engineered *Halomonas bluephagenesis* TD01

**DOI:** 10.3390/molecules26133778

**Published:** 2021-06-22

**Authors:** Jiping Zhao, Ganqiao Ran, Mengmeng Xu, Xiaoyun Lu, Dan Tan

**Affiliations:** 1 Key Laboratory of Biomedical Information Engineering of the Ministry of Education, Department of Biological Science and Bioengineering, School of Life Science and Technology, Xi’an Jiaotong University, Xi’an 710049, China; zjp93823@sina.com (J.Z.); yxmm11@stu.xjtu.edu.cn (M.X.); 2 Institute of Bio-Agriculture of Shaanxi Province, Xi’an 710043, China; ranganqiao@ms.xab.ac.cn

**Keywords:** L-DOPA, tyrosinase, *Halomonas bluephagenesis*, polyhydroxyalkanoates, immobilization

## Abstract

3,4-dihydroxyphenyl-L-alanine (L-DOPA) is a preferred drug for Parkinson’s disease, with an increasing demand worldwide that mainly relies on costly and environmentally problematic chemical synthesis. Yet, biological L-DOPA production is unfeasible at the industrial scale due to its low L-DOPA yield and high production cost. In this study, low-cost *Halomonas bluephagenesis* TD01 was engineered to produce tyrosinase TyrVs-immobilized polyhydroxyalkanoate (PHA) nanogranules in vivo, with the improved PHA content and increased immobilization efficiency of TyrVs accounting for 6.85% on the surface of PHA. A higher L-DOPA-forming monophenolase activity of 518.87 U/g PHA granules and an L-DOPA concentration of 974.36 mg/L in 3 h catalysis were achieved, compared to those of *E. coli*. Together with the result of L-DOPA production directly by cell lysates containing PHA-TyrVs nanogranules, our study demonstrated the robust and cost-effective production of L-DOPA by *H. bluephagenesis*, further contributing to its low-cost industrial production based on next-generation industrial biotechnology (NGIB).

## 1. Introduction

More than 10 million people worldwide are living with Parkinson’s disease (PD), a prevalent degenerative disorder of the central nervous system that affects the nerve cells in the brain with reduced dopamine levels. 3,4-dihydroxyphenyl-L-alanine (L-DOPA) is currently the most promising drug for PD treatment [1] since it can cross the blood–brain barrier and functions as the precursor of dopamine, alleviating Parkinson’s disease [2]. With increasing cases of PD in both the elderly and the younger generation, it is estimated that the annual demand for L-DOPA worldwide is increasing with a market size of 250 metric tons [3]. 

Currently, L-DOPA production mainly relies on chemical synthesis, as evident with the successful case of Monsanto company. It requires multistep procedures and harsh reaction conditions, resulting in various toxic intermediates and limited economic benefit as well as the inactive racemic DL-mixture [4,5]. Additionally, the direct extraction of L-DOPA from various legumes such as *Vicia faba* and *Mucuna pruriens* has commonly shown low yields and limited annual productivity with longer periods [6]. Substantial efforts have been made to synthetize L-DOPA biologically using a variety of biocatalysts such as tyrosine phenol-lyase (Tpl) [7], tyrosinase (Tyr) [8], or *p*-hydroxyphenylacetate 3-hydroxylase (PHAH) [7,9], either by microbial fermentation and biotransformation in vivo or biocatalytic conversion in vitro. 

Microbial fermentation has been extensively studied in various hosts such as *E. coli* [10], *Yarrowia lipolytica* [11], *Erwinia herbicola* [12], etc. with limited commercial success in Ajinomoto Co. Ltd. Due to the relatively higher productivity and simpler purification procedures compared to microbial fermentation, biocatalytic conversion, especially the simple tyrosinase catalytic procedure, has become the main focus in both research and industry [13]. Tyrosinase catalyzes the oxidation of *ortho*-hydroxylation of L-tyrosine to L-DOPA via its monophenolase (MP, EC 1.14.18.1) activity, and consecutively, diphenolase (DP, EC 1.10.3.2) activity for the further oxidation of L-DOPA to L-dopaquinone [14]. Screening tyrosinases that exhibit higher MP activity than DP activity and adding ascorbic acid as a reducing agent, will inhibit the subsequent conversion of L-DOPA to L-dopaqunione or L-dopachrome and increase L-DOPA accumulation [11].

As immobilization engineering will further increase the L-DOPA titer by promoting enzyme recycling and preventing enzyme denaturation [11], different immobilization strategies of tyrosinase have been uncovered [15,16,17]. Recently, a new nanobiocatalyst was prepared by immobilizing tyrosinase from *Verrucomicrobium spinosum* (TyrVs) [13], which exhibits a high MP activity on polyhydroxyalkanoate (PHA) nanogranules [18]. PHAs are insoluble intracellular nanogranules with diameters of 50–500 nm surrounded by numerous granule-associated proteins, including PHA synthases (PhaC), Phasins (PhaP), etc. in native producer [19,20]. By fusing TyrVs to PhaC, PHA nanogranules with active TyrVs on their surface were produced in *E. coli* in one-step simultaneously with PHA synthesis, avoiding tedious traditional immobilization procedures. Additionally, L-DOPA productivity of 148.70 mg/L/h with reasonable reusability was achieved by PHA nanogranules after 3 h enzymatic catalysis under optimized conditions [18]. However, the nanocatalyst produced in *E. coli* exhibited low PHA accumulation with the complicated purification procedure of PHA nanogranules, as well as low immobilized efficiency of TyrVs on the PHA surface, thus limiting further improvements of L-DOPA production. Although different immobilization methods for recycled tyrosinase have been explored, the productivity of L-DOPA has remained below 500 mg/L/h, far from the level of industrial production [4]; thus, a novel cost-effective host was urgently needed. 

*Halomonas bluephagenesis* TD01 is a fast-growing and contamination-resistant extremophilic PHA producer that has been demonstrated to be a low-cost industrial host with a unique open and continuous fermentation process for the next generation industrial biotechnology (NGIB) [21,22]. Industrial fermentations up to 5000 L scale have been successfully conducted using *H. bluephagenesis* TD in China, grown on low-cost substrates and seawater under open, nonsterile conditions with recycling of culture broth; conserquently, a high cell density of about 100 g/L cell dry weight (CDW), along with stable productivity of PHA, was achieved, demonstrating its good potential for industrial microbial production of chemicals [23].

**Figure 1:**
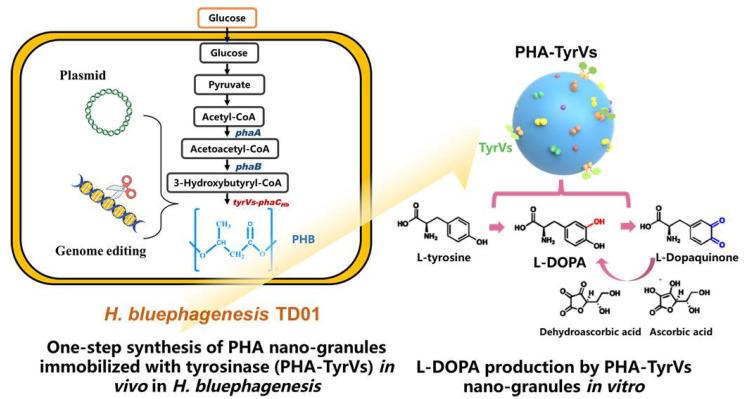
Engineering of *Halomonas bluephagenesis* TD strain for the production of TyrVs immobilized PHA nanogranules in vivo and the in vitro catalysis for L-DOPA production by PHA-TyrVs nanogranules. TyrVs-PhaC_Hb_ fusion protein was expressed in plasmid or integrated expressed in genome in *H. bluephagenesis* TD strain, and the PHA-TyrVs nanogranules were produced with TyrVs-PhaC_Hb_ protein displayed onto the surface of PHA simultaneously with native PHA synthesis from glucose. The biocatalyst was purified and subjected to efficient L-DOPA production in vivo in the presence of ascorbic acid to inhibit further oxidation.

This study focuses on the engineering of the industrial host *H. bluephagenesis* TD01, aiming to promote high-yield and low-cost production of L-DOPA. Tyrosinase-immobilized PHA nanogranules were produced in one step via plasmid expression or genome-integrated expression in vivo in an engineered *H. bluephagenesis* TD strain, and subsequent in vitro biocatalysis was performed using purified PHA nanogranules or cell lysates for the L-DOPA production (Figure 1).

## 2. Results

### 2.1. Construction and Identification of PhaC_Hb_ Knockout Mutant H. bluephagenesis TDΔC

**Figure 2:**
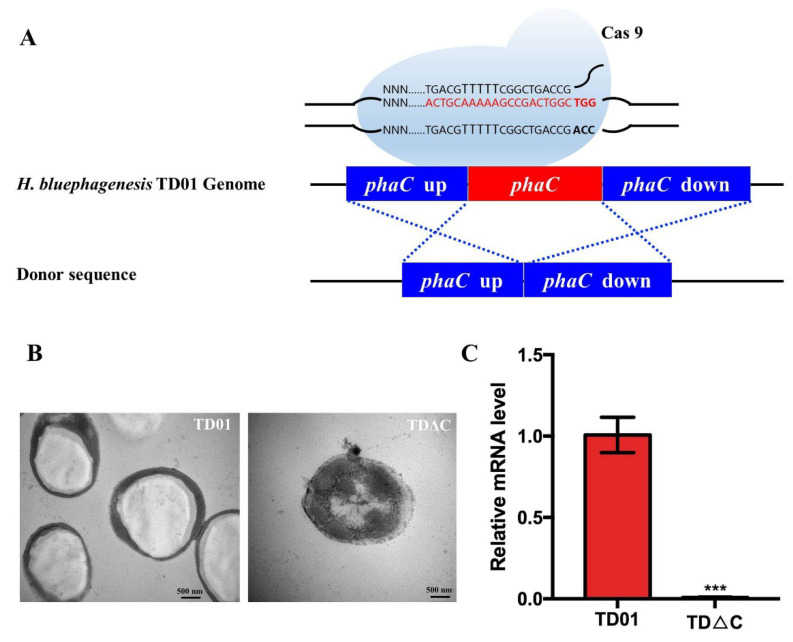
Identifications of PhaC_Hb_ knockout mutant *H. bluephagenesis* TDΔC: (**A**) PhaC_Hb_ knockout procedure by CRISPR-Cas 9 method using 500-bp homologous arm upstream and downstream of the *phaC_Hb_* gene; (**B**) TEM observation of wild-type *H. bluephagenesis* TD01 (left) and *H. bluephagenesis* TDΔC (right). Bar: 500 nm; (**C**) Relative mRNA level of *phaC_Hb_* gene in wild-type and knockout mutants determined by RT-qPCR. *** means *p* < 0.001.

Wild-type *H. bluephagenesis* TD01 is able to accumulate PHA to up to 80 wt% of cell dry weight [24]. To avoid the competitive consumption of energy and carbon source of native PHA synthesis for L-DOPA production, the PHA synthase (PhaC_Hb_) knockout strain *H. bluephagenesis* TDΔC was constructed using an adapted CRISPR/Cas 9 method reported previously [25]. Briefly, 500-base-pair (bp) homologous arms were employed to achieve higher knockout efficiency (Figure 2A). The knockout mutants were verified by colony PCR using the designed primers upstream and downstream of the *phaC_Hb_* gene, and almost 100% of the randomly selected clones were knockouts, which were further confirmed by sequencing (Figure S1). As shown in Figure 2C, compared to the wild type, no expression of PhaC_Hb_ was detected in *H. bluephagenesis* TDΔC at the mRNA level by RT-qPCR. Additionally, the cell was observed to be filled with a single large granule by transmission electron microscopy (TEM) in the wild type, while no PHA granules were found in knockout mutants, confirming the loss of PhaC_Hb_ function.

### 2.2. Expression of Free Tyrosinase TyrVs in H. bluephagenesis TD Strain and Determination of Its L-DOPA Productivity 

**Figure 3:**
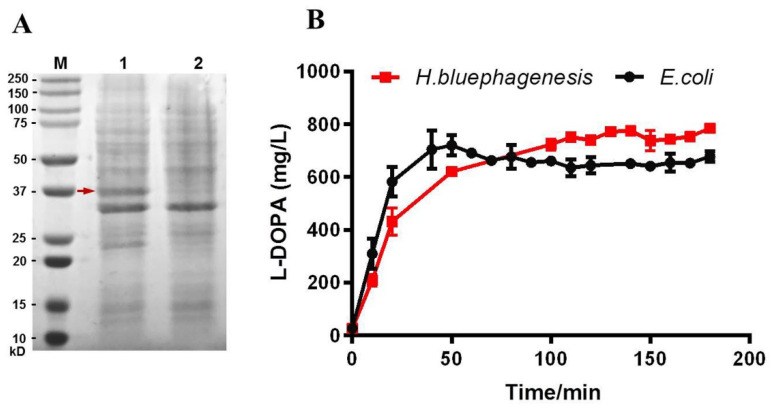
Expression of free TyrVs in *H. bluephagenesis* TD strain and L-DOPA productivity in recombinant *H. bluephagenesis* TD and *E. coli* harboring free TyrVs: (**A**) expression of free TyrVs in recombinant *H. bluephagenesis* TD (pMCS1-Vs) was confirmed by SDS-PAGE of whole-cell lysates. Lane 1, *H. bluephagenesis* TD (pMCS1-Vs), Lane 2, wild-type *H. bluephagenesis* TD, M, protein marker. The red arrow indicates the 37 kD band of free TyrVs; (**B**) L-DOPA production by the same volume of cell lysates of *H. bluephagenesis* and *E. coli* harboring free TyrVs. The error bars indicate the SD values from triplicate trials.

Expression of free TyrVs was initially assessed in *H. bluephagenesis* TD strain to test its expression level and L-DOPA productivity in *H. bluephagenesis*. As shown in Figure 3A, a 37 kD protein band can clearly be observed when the whole-cell lysates of recombinant *H. bluephagenesis* TDΔC (pMCS1-Vs) were subjected to SDS-PAGE, suggesting that TyrVs is efficiently expressed in *H. bluephagenesis* strain. 

As much as 5 mM L-tyrosine, which is the highest substrate concentration reported for intracellular L-DOPA synthesis, was then added to test the L-DOPA productivity of the recombinant *H. bluephagenesis* TD strain in vivo, and unfortunately, no L-DOPA was detected in either cells or supernatant. The same result was also observed in recombinant *E. coli* BL21 (pCDF-Vs) (data not shown). The failure of L-DOPA synthesis in vivo may be attributed to the poor transportation of tyrosine into both host cells, leading to the limited intracellular substrate concentration [7]. 

Therefore, an in vitro enzymatic catalysis by cell lysates was then conducted to provide enough substrate. The same volume (150 μL) of cell lysates of *H. bluephagenesis* TDΔC (pMCS1-Vs) and *E. coli* BL21 (pCDF-Vs) was directly subjected to enzymatic reaction in vitro using 2.5 mM L-tyrosine in the presence of 5 mM ascorbic acid and 1 μM Cu_2_SO_4_, which act as a reducing agent [11] and cofactor, respectively. As shown in Figure 3B, in both cell-lysate catalysis reactions, the accumulations of L-DOPA increased rapidly in the first 50 min and gradually remained stable after that in the 3 h catalysis. The cell lysates of the *H. bluephagenesis* TD strain achieved a consistently higher concentration of L-DOPA after 50 min, reaching 784.61 mg/L after 3 h catalysis, compared to 677.69 mg/L of *E. coli*. These results clearly show the stable and improved L-DOPA production in *H. bluephagenesis*, indicating that it is a desirable host for L-DOPA production. 

### 2.3. One-Step Production and Identification of PHA-TyrVs Nanogranules by H. bluephagenesis

**Figure 4:**
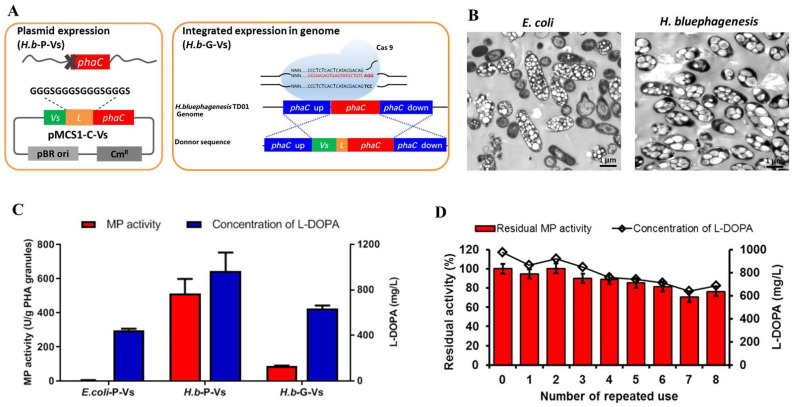
Production and characterization of PHA-TyrVs nanogranules produced by *H. bluephagenesis*: (**A**) plasmid expression and integrated expression of *tyrVs-phaC_Hb_* fusion gene in *H. bluephagenesis*, to generate two PHA nanogranules producers, *H.b*-P-Vs and *H.b*-G-Vs, respectively. The *tyrVs* gene was fused to the 5′end of *phaC_Hb_* gene via a 16-amino-acid linker; (**B**) TEM observation of PHA nanogranules in the cell of *E. coli* and *H. bluephagenesis* after cultured for 48 h. Bar: 1 μm; (**C**) comparison of monophenolase (MP) activity and L-DOPA yield of purified PHA-TyrVs nanogranules produced in three hosts. Moreover, 1 mL enzymatic reaction was performed under the optimized condition of 2.5 mM L-tyrosine, 5 mM ascorbic acid, 1 μM Cu^2+^, and 1.5 mg/mL PHA-TyrVs nanogranules, pH 6.0 and 50 °C, rotating at 200 rpm for 3 h. (**D**) Reusability of PHA-TyrVs nanogranules produced by *H. bluephagenesis H.b*-P-Vs that has the highest content of TyrVs immobilized on PHA surface. After one cycle of 3 h reaction for L-DOPA synthesis, the PHA-TyrVs nanogranules were recycled and resuspended in the same reaction mixture and repeated for eight rounds. The initial activity was set to 100%.

PHA-TyrVs nanogranules, as a novel immobilization strategy of tyrosinase, were attempted in halophilic *H. bluephagenesis*. The TyrVs was N-terminally fused to PhaC_Hb_ and expressed in plasmid to complement the loss of *phaC_Hb_* in the genome of *H. bluephagenesis* TDΔC, as well as to produce TyrVs-decorated PHA nanogranules in vivo. Recombinant *H. bluephagenesis* TDΔC (pMCS1-C-Vs) (termed *H.b*-P-Vs) with *tyrVs-phaC_Hb_* fusion gene expressed in plasmid achieved 2.50 g/L CDW with 23.22 wt% PHA content in cells after 48 h cultivation (Table 1), which was also confirmed by TEM in Figure 4B. The result indicated that PhaC retained an intact ability to synthesize PHA even after N-terminal fusion with TyrVs. 

To improve the intracellular synthesis of PHA nanogranules and to simplify the purification procedures, it is favorable to express the expression cassette for PHA synthesis in genome without adding antibiotics. Therefore, the *tyrVs-phaC_Hb_* fusion gene was introduced into the genome of *H. bluephagenesis* TD01 by the CRISPR/Cas9 method (Figure 4A). The *tyrVs* gene with a linker sequence was fused to the 5′ end of *phaC_Hb_* and the cassette was knocked into the genome via 500 bp homologous arm up and downstream of the *phaC_Hb_*, respectively (Figure 4A). Colony PCR (Figure S1b) and sequencing results confirmed that a longer fragment was integrated into the *phaC_Hb_* loci of *H. bluephagenesis* TD01 genome, replacing *phaC_Hb_* with *tyrVs-phaC_Hb_*. The mutant strain named “*H.b*-G-Vs” was able to grow to 9.64 g/L and accumulated PHA nanogranules to up to 56.10 wt% of cell dry weight, as determined by GC analysis and TEM observation (Table 1 and Figure 4B), resulting in 5.41 g/L PHA nanogranules. The results demonstrated the significant improvement in cell growth and PHA synthesis after integrated expression of *tyrVs-phaC_Hb_* in the genome, compared to its plasmid expression in the *H.b*-P-Vs strain (Table 1). Specifically, regardless of whether the TyrVs-PhaC_Hb_ fusion protein was expressed in the plasmid (*H.b*-P-Vs) or genome (*H.b*-G-Vs), the cell growth and/or PHA content were greatly improved, compared to those of *E. coli*, and more than 20-fold increased concentration of PHA was achieved in *H. bluephagenesis* (Table 1), revealing the advantages of *H. bluephagenesis* as a good host for the synthesis of PHA nanogranules.

The PHA-TyrVs purified from cells of *H.b*-P-Vs or *H.b*-G-Vs was subjected to LC-MS/MS analysis for absolute quantification of immobilized TyrVs on the surface of PHA nanogranules, as previously described [18]. In total, more than 270 proteins could be confidently identified (protein score >30, FDR <1%) on the surface of the PHA granules, among which TyrVs-PhaC_Hb_ was dominant. As shown in Supplementary Table S2 and Figure S3, immobilized TyrVs in *H.b*-P-Vs was calculated to occupy approximately 6.85% of the total weight of the purified PHA nanogranules [6.85% = (1/0.0146) (light/heavy) × (1:1000) (the ratio of dilution, light/heavy)] (Table S2). It is more than 100 fold higher than that of *E. coli*, which was approximately 0.06% (Table 1 and Figure S2) [18]. Meanwhile, TyrVs immobilized on the surface of PHA by integrated expression in the genome of *H.b*-G-Vs accounted for about 3.48% (Table S2 and Figure S4), which is still higher than that of *E. coli* but slightly lower than that of *H.b*-P-Vs strain, as the TyrVs-PhaC_Hb_ fusion protein was expressed in a higher-copy-number plasmid in *H.b*-P-Vs. The PHA-TyrVs nanogranules produced by *H. bluephagenesis* exhibited significantly increased content and immobilized TyrVs, which would benefit its activity and further synthesis of L-DOPA.

### 2.4. L-DOPA Production and Reusability of PHA-TyrVs Nanogranules Produced by H. bluephagenesis

The purified PHA-TyrVs nanogranules produced by *H. bluephagenesis* were subjected to a 1 mL-enzymatic reaction to test their L-DOPA productivity under optimized conditions. As shown in Figure 4C, the TyrVs immobilized on the PHA granules in *H.b*-P-Vs exhibited a specific MP activity of 518.87 U/g PHA granules, about 94-fold that of *E. coli*, which was only 5.49 U/g PHA granules. The remarkably increased activity resulted in an L-DOPA concentration of 974.36 mg/L, compared to 446.10 mg/L in *E. coli*. Additionally, the PHA-TyrVs produced by genome integrated expression in *H.b*-G-Vs achieved a specific MP activity of 86.60 U/g PHA granules and an L-DOPA concentration of 633.90 mg/L (Figure 4C). The MP activity of tyrosinase and the L-DOPA concentration in *E. coli*-P-Vs and *H.b*-G-Vs were reasonably lower than that in the *H.b*-P-Vs strain due to the lower content of immobilized TyrVs on the PHA surface (Table 1 and Figures S2–S4). Our results clearly demonstrate the remarkable improvement in activity and L-DOPA productivity of *H. bluephagenesis*-produced PHA-TyrVs, especially in the plasmid expression strain *H.b*-P-Vs, compared with the traditional host *E. coli* that is not a native producer of PHA. 

The reusability of the PHA-TyrVs nanogranules produced by *H.b*-P-Vs strain was also tested by repeated use of nanogranules for eight cycles. As shown in Figure 4D, the MP activity remained stable and retained more than 76% of its initial activity after repeated uses, leading to a relatively stable concentration of L-DOPA. The good reusability of *H. bluephagenesis*-produced PHA-TyrVs will be beneficial for the cost-effective production of L-DOPA.

### 2.5. Robust L-DOPA Production by Cell Lysates Containing PHA-TyrVs Nanogranules from Three Hosts

**Figure 5:**
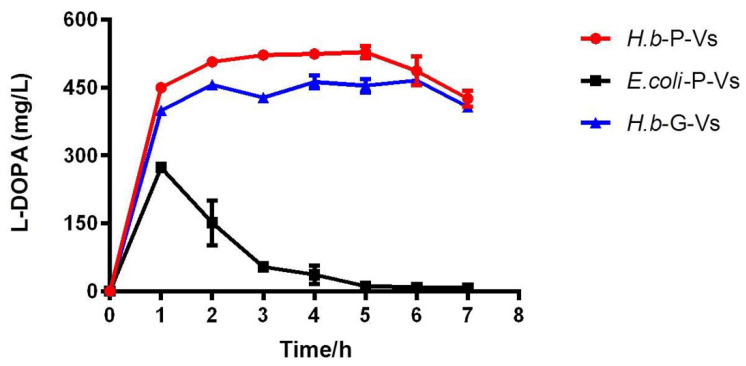
Direct catalysis by cell lysates containing PHA-TyrVs nanogranules from three hosts for L-DOPA production. The cells of three hosts (*E. coli*-P-Vs, *H.b*-P-Vs, *H.b*-G-Vs) cultured for 48 h were ultrasonically disrupted, and the same volume of cell lysates (150 μL) was added to produce L-DOPA in 1 mL scale enzymatic reaction, incubated at 50 °C, at 200 rpm lasting for 7 h, with sampling every 1 h. Error bars indicate SD values from triplicate trials.

The high content of PHA granules in the cells of *H. bluephagenesis* makes it possible for direct catalysis by cell lysates to avoid the complicated purification procedure of PHA nanogranules. Thus, a primary test for L-DOPA production by cell lysates from three hosts containing PHA-TyrVs nanogranules was conducted. The same volume of cell lysates (150 μL) from three hosts (*E. coli*-P-Vs, *H.b*-P-Vs, and *H.b*-G-Vs) was added to produce L-DOPA in 1 mL scale enzymatic reaction, lasting for 7 h, with sampling every 1 h. As shown in Figure 5, the synthesis of L-DOPA increased rapidly in the first 1 h for all three cell lysates, among which the concentrations of L-DOPA produced by two *H. bluephagenesis* strains were obviously higher than that in *E. coli*. Additionally, the production of L-DOPA in *H.b*-P-Vs and *H.b*-G-Vs stably increased and was maintained at a level of above 450 mg/L after 1 h, and the highest L-DOPA concentrations in *H.b*-G-Vs and *H.b*-P-Vs were 465.55 and 528.30 mg/L, respectively. In contrast, the L-DOPA concentration of *E. coli*-P-Vs reached a maximum value of 273.38 mg/L and decreased rapidly after that, remaining at nearly zero in 7 h. The great differences in L-DOPA production between *E. coli* and *H. bluephagenesis* may be attributed to the reduced PHA content and TyrVs immbolization efficiency on the PHA granules in the same volume of cell lysates (Table 1). The results also revealed that 3–5 h is adequate for stable and high-yield production of L-DOPA in *H. bluephagenesis* strains. Our primary test for L-DOPA synthesis by cell lysates revealed the robustness and high efficiency of *H. bluephagenesis* as a host.

## 3. Discussions

Tyrosinase-based biocatalytic conversion for L-DOPA production has become the main focus in both research and industry. To address the bottlenecks of biological L-DOPA production scale up from lab to industry, the *H. bluephagenesis* TD strain, which can be used for low-cost NGIB process, was engineered in our study to produce L-DOPA based on a novel immobilization strategy- using PHA-TyrVs nanogranules. 

The PHA synthetase PhaC_Hb_ in the genome of *H. bluephagenesis* was knocked out by CRISPR/Cas 9 and complementarily expressed as the TyrVs-PhaC_Hb_ fusion protein to facilitate the synthesis of TyrVs-decorated PHA nanogranules in vivo. The rapid, efficient, and scarless CRISPR/Cas 9 system, especially applicable in *H. bluephagenesis* TD01 comprises a low-copy-number plasmid expressing Cas9 and a high-copy-number plasmid equipped with a sgRNA template and donor DNA insert to avoid linear DNA transformation by electroporation, which is difficult to achieve in the *H. bluephagenesis* TD strain [25]. High efficiency of the knockout was achieved, and the PhaC_Hb_ knockout mutant was demonstrated to be a good platform for PHA nanogranules production. 

The TyrVs-PhaC_Hb_ fusion protein was expressed in the plasmid (*H.b*-P-Vs) or in the genome (*H.b*-G-Vs) in *H. bluephagenesis*, and one-step immobilization of TyrVs on the surface of PHA-TyrVs nanogranules was achieved simultaneously with PHA synthesis, thus avoiding tedious traditional immobilization procedures. Significantly increased TyrVs immobilization accounting for 6.85% and 3.48% on the PHA surface in *H.b*-P-Vs and *H.b*-G-Vs, respectively, was obtained, compared to that of *E. coli*, resulting in higher MP activities and L-DOPA productivities as shown in Figure 4C and Figure 5. The increase in TyrVs immobilization on the surface of PHA granules in *H. bluephagenesis* may be attributed to the synthesis of PHA and the anchoring of TyrVs-PhaC_Hb_ fusion protein on PHA nanogranules being properly regulated in the native PHA producer *H. bluephagenesis*. Other PHA-associated proteins in *H. bluephagenesis* such as PHA phasin protein PhaP, etc., which is absent in *E. coli*, may help to form the stable structure of PHA nanogranules, benefiting the enzyme immobilization [19,20]. In fact, the TyrVs-PhaC expression in *E. coli* was extremely higher than that of *H. bluephagenesis*, as determined by RT-qPCR (data not shown), because of the higher-copy-number of plasmid used in *E. coli* [18], indicating that the TyrVs-PhaC fusion proteins were highly expressed, while the relatively unstable structure of PHA granules may inhibit their immobilization on PHA nanogranules in *E. coli*. The stable structure of PHA-TyrVs nanogranules also leads to a higher content of PHA and a larger size of PHA granules in cells of *H. bluephagenesis* than those of *E. coli*, which can be observed in the TEM in Figure 4B. The significantly increased TyrVs immobilization and increased accumulation of PHA-TyrVs produced by *H. bluephagenesis* also benefit its activity and further synthesis of L-DOPA.

The purpose of integrated genome expression of TyrVs-PhaC_Hb_ fusion protein in *H.b*-G-Vs is to address the poor cell growth and low PHA content in the plasmid-expressed version in *H.b*-P-Vs, which is caused by the addition of antibiotics to maintain the plasmid in cells. Although our results showed that the PHA-TyrVs nanogranules produced in the *H.b*-G-Vs strain are less satisfactory, compared to those in the *H.b*-P-Vs strain, the remarkably increased cell growth and content of PHA will facilitate the purification process and increase the intracellular concentration of PHA-TyrVs nanogranules, also contributing to further L-DOPA production. The lower content of immobilized TyrVs in the *H.b*-G-Vs strain can be addressed in the future using multiple integrations and higher-copy-number expression in the genome.

Furthermore, a primary study on direct catalysis by *H. bluephagenesis* cell lysates containing PHA-TyrVs nanogranules for L-DOPA production was also conducted, as most of the cell lysates were TyrVs-decorated PHA nanogranules, thus avoiding the complicated purification procedures. A stably increasing L-DOPA production was achieved by cell lysates of *H. bluephagenesis*, compared to that of *E. coli*, demonstrating their robustness. With optimizations in the future, the PHA content and concentration can be further improved, allowing a more cost-effective production of L-DOPA by *H. bluephagenesis* cell lysates.

Our study demonstrated that *H. bluephagenesis* is a great host for the production of PHA nanogranules due to its increased cell growth and PHA accumulation, which will simplify the downstream purification process. The relatively larger size of PHA granules in cells (Figure 4B) will also facilitate centrifugation and downstream processing [26]. Furthermore, the remarkably improved content of immobilized TyrVs on the surface of PHA nanogranules benefits their higher activity and higher L-DOPA productivity, making it an efficient host for L-DOPA production by PHA nanobiocatalyst. In addition, the intracellular ectoine in *Halomonas*, an amino acid derivative mainly responsible for maintaining osmotic pressure [27], has also been reported to help improve the stability and activity of enzymes [28,29], contributing to more stable tyrosinase activity as well as higher L-DOPA productivity in *H. bluephagenesis*. The advantages of *H. bluephagenesis* in the synthesis of PHA nanogranules will also facilitate the easy immobilization of other enzymes, which will avoid the tedious traditional immobilization procedures.

The highest L-DOPA productivity of 324.79 and ~176.10 mg/L/h for pure PHA-TyrVs catalysis and cell-lysates catalysis, respectively, were achieved in our study, both of which are comparably higher than the value of 146.7 mg/L/h in *E. coli* [18]. Compared with other studies on L-DOPA production by biocatalytic conversion of immobilized tyrosinase with the highest productivity of 209.0 mg/L/h, our productivity also reached a remarkable level but needs further improvement using more synthetic biology engineering of *H. bluephagenesis* as well as culture condition optimizations [7,8,30,31]. 

## 4. Materials and Methods

### 4.1. Microorganisms, Plasmids, and Culture Conditions

The plasmids and bacterial strains used in this study are listed in Table 2. *H. bluephagenesis* TD01 (public collection No. CGMCC4353) and derivative strains were grown in 60 LB medium (10 g/L tryptone, 5 g/L yeast extract, and 60 g/L NaCl) at 37 °C, 200 rpm [24]. *E. coli* S17-1 pir was used as a vector donor for plasmid transformation to *H. bluephagenesis* TD01 via conjugation. *E. coli* cells were cultured in LB medium at 37 °C and 200 rpm on a rotary shaker. A broad host plasmid pBBR1MCS-1 [32] was used as a vector backbone for gene expression and PHA nanogranules production in *H. bluephagenesis* strain. Furthermore, pQ08 expressing Cas9 from *Streptococcus pyogenes* and pQ31 with a sgRNA template and donor DNA were used to construct the CRISPR/Cas9 system for gene knockout and knockin [25]. Chloramphenicol (Sangon Biotech, Shanghai, China) or streptomycin (Reyoung Company, Zibo, China) was added at a concentration of 25 μg/mL or 100 μg/mL, respectively, where appropriate.

### 4.2. Molecular Manipulations of H. bluephagenesis TD Strain

All the DNA manipulations in this study were based on standard protocols or manufactures’ instructions, and the primers used are listed in Table S1. Plasmid extraction and DNA purification kits were purchased from Qiangen (Shanghai, China). All DNA oligonucleotides were obtained from GENEWIZ (Suzhou, China). Plasmids were transformed from *E. coli* S17-1pir to *H. bluephagenesis* TD strains via the conjugation method, as described previously [34]. 

#### 4.2.1. Construction of Expression Vectors for Free and Immobilized TyrVs in *H. bluephagenesis* TD Strain 

To construct the expression vector of free TyrVs, the tyrosinase gene *tyrVs* from *Verrucomicrobium spinosum* (GeneBank No. WP_081452337) was amplified and cloned into the medium-copy-number vector pBBRMCS-1 under the control of constitutive strong P_porin_ promoter to generate pMCS1-Vs [34]. 

Regarding the construction of the expression vector for the production of PHA nanogranules decorated with TyrVs in *H. bluephagenesis*, the tyrVs gene with a linker sequence (amino acid GGGSGGGSGGGS), as reported previously [18], was fused to the 5′ end of the *phaC_Hb_* gene of *H. bluephagenesis* to generate *tyrVs-phaC_Hb_* fusion gene. In detail, the *tyrVs* gene and a linker sequence were amplified from pCDF-ABC-Vs [18] using the primer pairs Vs-MCS-F and Vs-MCS-R. Additionally, the *phaC_Hb_* gene (class I PHA synthase from *H. bluephagenesis* TD01, GeneBank No. EGP20415.1) without the start codon was amplified from the genome of *H. bluephagenesis* TD01 using the primers phaC-Hb-F and phaC-Hb-R. Three fragments of P_porin_ promoter, *tyrVs-linker*, and *phaC_Hb_* were subsequently ligated to *Sac* I and *Xho* I digested pBBR1MCS-1 backbone using the Gibson assembly method [35], yielding pMCS1-C-Vs. 

#### 4.2.2. Genome Editing by Adapted CRISPR/Cas9 in *H. bluephagenesis* TD01

An adapted CRISPR/Cas 9 approach for *H. bluephagenesis* TD01 was employed for gene knockout and knockin [25]. For *phaC_Hb_* knockout, 500 bp homologous arms (HR) upstream and downstream of the *phaC_Hb_* gene as the donor DNA were amplified from the genome of *H. bluephagenesis* TD01. The DNA sequence for guide RNA was selected near a PAM site and amplified from the genome. The donor DNA and DNA sequence of guide RNA were digested with *Bsa* I and subsequently ligated to the linear backbone plasmid pQ31 via the Golden Gate method [36], generating pQ31-C. To achieve genome-integrated expression of immobilized tyrosinase, the *tyrVs-phaC_Hb_* fusion gene with a linker sequence constructed above was knocked into the *H. bluephagenesis* genome. Similarly, 500 bp homologous arms upstream and downstream of the *phaC_Hb_* were added to the *tyrVs-phaC_Hb_* fragment, which served as the donor DNA. Then, the donor DNA and the DNA sequence of guide RNA was ligated to the linear backbone plasmid pQ31 to construct pQ31-VsC for knockin. Genome editing was performed by sequentially transferring the Cas9-expressing plasmid pQ08 and sgRNA+donor DNA providing plasmid pQ31-C or pQ31-VsC into *H. bluephagenesis* TD01 via conjugation. The Cm^R^ and Spe^R^ clones were then selected to further identify the knockout or knockin mutants by colony PCR using the primers C-test-F and C-test-R (Table S1). 

### 4.3. Production and Purification of PHA-TyrVs Nanogranules 

The production of PHA nanogranules in the *H. bluephagenesis* TD01 was described previously using a modified MMG medium with 60 g/L NaCl and 20 g/L glucose [37]. The PHA nanogranules were isolated from the cell lysate using ultracentrifugation on a glycerol gradient [38] and washed 3 times with 10 volumes of 50 mM PBS. Finally, PHA nanogranules were resuspended in 50 mM PBS buffer and lyophilized, thus yielding purified PHA powder. 

### 4.4. Characterizations of PHA-TyrVs Nanogranules

#### 4.4.1. PHA Content Determination and Granule Visualization 

The PHA content in the cell was determined by gas chromatography (GC) based on a previous study [24]. Bacteria were harvested after 48 h cultivation via centrifugation at 10,000× *g* for 10 min and washed with deionized water once. Cell dry weights (CDW) were measured after lyophilization and PHA content was analyzed using GC (GC-500, PerkinElmer, USA) after methanolysis of the lyophilized cells [24]. Cells were harvested by centrifugation at 10,000× *g* for 5 min, and then the precipitated cells were prepared for transmission electron microscopy (TEM, HitachiH-7650B, Tokyo, Japan) analysis as described in [39].

#### 4.4.2. Identification and Absolute Quantification of Immobilized TyrVs on PHA Nanogranules via MS-Based Quantitative Proteomic

Mass spectrometric characterization of proteomes was employed in this study to identify and quantify the possible proteins on the surface of our PHA-TyrVs nanogranules, as reported previously [18,40,41]. The isobaric dimethylation was used to label the proteins before being subjected to LC-MS/MS analysis on a NanoLC system (UltiMate 3000 RSLC nanoLC system, AB SCIEX, Framingham, MA, USA). The raw data were processed using Maxquant software for protein identification and quantification. A customized database was used to enable accurate protein identification and quantification [18].

### 4.5. Production of L-DOPA 

#### 4.5.1. Production of L-DOPA In Vivo

The culture medium for intracellular L-DOPA synthesis contained 7 g/L yeast extract, 7.5 g/L (NH_4_)_2_SO_4_, 2 g/L KH_2_PO_4_, 3 g/L K_2_HPO_4_·3H_2_O, 1 g/L MgSO_4_·7H_2_O, 10 g/L glucose, 5 g/L glycerol, 0.45 g/L ascorbic acid, and 1 μM CuSO_4_ [3]. In addition, 0.5 mM, 1 mM, and 5 mM substrate L-tyrosine were added. *E. coli* BL21(DE3) harboring pCDF-Vs and *H. bluephagenesis* harboring pMCS1-Vs were grown at 37 °C for 12 h, and then the cells were transferred to the above culture medium for the synthesis of L-DOPA in vivo. A total of 1 mM IPTG was added after 3 h in *E. coli* to promote TyrVs expression under the T7 promoter. Cells were harvested after 24 h cultivation and the pellet and the supernatant were collected for L-DOPA detection. 

#### 4.5.2. Production of L-DOPA In Vitro by Cell Lysates or Purified PHA-TyrVs Nanogranules

*H. bluephagenesis* TD strain harboring pMCS1-Vs and *E. coli* BL21(DE3) harboring pCDF-Vs were cultured for 24 h in 60 LB or LB medium, respectively, and cells were collected and washed once with 50 mM PBS (pH 6.0), suspended in 50 mM PBS at a ratio of 10:1, and then ultrasonically disrupted for 5 s and stopped for 5 s (power was 400 W, and time was 30 min). Then, 150 μL cell lysates were added in 1 mL scale enzymatic reaction to produce L-DOPA in PBS buffer containing 2.5 mM L-tyrosine, 5 mM ascorbic acid, and 1 μM Cu_2_SO_4_. The reaction was performed in an incubator shaker at 50 °C and 200 rpm [18], lasting for 3 h with sampling every 10 min. The same procedure was conducted for direct catalysis by cell lysates from three hosts harboring PHA-TyrVs nanogranules (*E. coli*-P-Vs, *H.b*-P-Vs, and *H.b*-G-Vs), which lasted for 7 h with sampling every 30 min.

For the catalysis by purified PHA-TyrVs nanogranules, the same reaction was performed under the above optimal conditions, using 1.5 mg/mL purified PHA-TyrVs nanogranules. The PHA granules without TyrVs immobilization served as a blank control. Samples were withdrawn to monitor the concentration of L-DOPA every 10 min. The reusability of PHA-TyrVs nanogranules for L-DOPA production was also assessed over eight rounds of repeated use, with each round lasting for 3 h [18]. For all in vitro reactions for L-DOPA synthesis, the control experiments leaving L-tyrosine in the same reaction mixture without the enzyme were also conducted to assess the spontaneous oxidation of L-tyrosine in the air.

### 4.6. Determination of L-DOPA Concentration and the Specific Monophenolase (MP) Activity of Tyrosinase 

The L-DOPA concentration was determined using Arnow’s method, as previously described [42]. The specific MP activity of tyrosinase was determined by the rate of L-DOPA formation from L-tyrosine in the presence of excess ascorbic acid, as measured via the A_460nm_ after treatment with Arnow’s reagent. One unit was defined as the amount of enzyme that catalyzes the formation of 1 μmol of L-DOPA per minute [18]. All measurements were conducted in triplicate.

## 5. Conclusions

To address the bottlenecks of upscaling biological L-DOPA production from lab scale to the industrial scale, a low-cost *H. bluephagenesis* TD strain was engineered to produce PHA-TyrVs nanogranules, and remarkably increased immobilization efficiency of TyrVs, a higher L-DOPA-forming MP activity, and L-DOPA productivity with good reusability were achieved, compared to those of *E. coli*. Together with the result of a primary study on direct L-DOPA production by cell lysates containing PHA-TyrVs granules, our study demonstrated the robust and cost-effective production of L-DOPA by *H. bluephagenesis,* further contributing to its low-cost industrial production based on NGIB.

## Figures and Tables

**Table 1:** Characteristics of PHA-TyrVs nanoparticles produced in three hosts.

Source of PHA-TyrVs Nanogranules	Plasmid Expression in *E. coli* (*E.coli*-P-Vs)	Plasmid Expression in *H. bluephagenesis* TD (*H.b*-P-Vs)	Genome Expression in *H. bluephagenesis* TD (*H.b*-G-Vs)
CDW (g/L)	1.14 ± 0.05	2.50 ± 0.17	9.64 ± 0.53
Content of PHA nanogranules in cell (wt%)	22.28 ± 1.25	23.22 ± 0.40	56.10 ± 2.60
Theoretical concentration of PHA nanogranules (g/L)	0.25 ± 0.01	0.58 ± 0.06	5.41 ± 1.12
MS quantification of immobilized content of TyrVs on PHA nanogranules	0.06%	6.85%	3.48%

**Table 2:** Plasmids and bacterial strains used in this study.

Strains/Plasmids	Descriptions	Sources/References
**Strains**		
*E. coli* DH5α	F-*φ-5dlacZ*Δlac Δ(*lacZYA-argF*)U169 *deoR recA1 endA1 hsdR17*(*rK*+,*mk*+) *phoA supE44 λ-thi-1 gyrA96 relA1*	Takara
*E. coli* BL21 (DE3)	F^-^ *ompT hsdS*_B_ (r_B_^-^ m_B_^-^) *gal dcm* (DE3)	Novagen
*E. coli* S17-1 pir	*TpR SmR recA*, *thi*, *pro*, *hsdR-M+RP4: 2-Tc:Mu: Km Tn7 λpir*	[33]
*H. bluephagenesis* TD01	*H. bluephagenesis* TD01 wild type, isolated from a salt lake in China	[24]
*H. bluephagenesis* TDΔC	*H. bluephagenesis* TD01 with *phaC_Hb_* gene deleted	This study
*H. bluephagenesis* *H.b*-G-Vs	*H. bluephagenesis* TD01 with *tyrVs*-*phaC_Hb_* fusion gene knocked in	This study
**Plasmids**		
pQ08	pSEVA321 derivative, *Streptococcus pyogenes cas9*, Cm^R^	[25]
pQ31	pSEVA241 derivative, P_J23119_-sgRNA template, Km^R^ and Spe^R^	[25]
pQ31-C	pQ31 derivative, P_J23119_-sgRNA template, 1000 bp donor DNA for *phaC_Hb_* knockout, Km^R^ and Spe^R^	This study
pQ31-VsC	pQ31 derivative, P_J23119_-sgRNA template, 3001 bp donor DNA for *tyrVs*-*phaC_Hb_* knockin, Km^R^ and Spe^R^	This study
pBBR1MCS-1	A broad host plasmid for *H. bluephagenesis* TD01, Km^R^	[32]
pMCS1-Vs	pBBR1MCS-1 derivative, P_porin_ promoter, free tyrosinase TyrVs expression vector in *H. bluephagenesis* TD, Cm^R^	This study
pMCS1-C-Vs	pBBR1MCS-1 derivative, P_porin_ promoter, expression vector for PHA-TyrVs nanogranules in *H. bluephagenesis* TD, Cm^R^	This study
pCDF-Vs	Expression vector for free tyrosinase from *Verrucomicrobium spinosum* TyrVs in *E. coli*, Sm^R^	[18]

## Data Availability

The data presented in this study are available in the article and Supplementary Materials.

## Supplementary Materials

The following are available online, Table S1: The sgRNA sequences and primers used in this study, Table S2: Result of mass spectrometer-based quantitative proteomics, Figure S1: The identification of PhaC_Hb_ knockout, Figure S2: Absolute quantification of fused TyrVs on surface of PHA-TyrVs nanogranules produced by *E. coli*-P-Vs using mass spectrometer-based quantitative proteomics, Figure S3: Absolute quantification of fused TyrVs on surface of PHA-TyrVs nanogranules produced by *H.b*-P-Vs using mass spectrometer-based quantitative proteomics, Figure S4: Absolute quantification of fused TyrVs on surface of PHA-TyrVs nanoparticles produced *H.b*-G-Vs using mass spectrometer-based quantitative proteomics.

## Sample Availability

Samples of the bacteria are available from the authors.
