# Supplementary material for: Cost-Effective Production of L-DOPA by Tyrosinase-Immobilized Polyhydroxyalkanoate Nanogranules in Engineered Halomonas bluephagenesis TD01
Source: Molecules. 2021 Jun 22;26(13):3778. doi: 10.3390/molecules26133778 (PMC8270294; doi:10.3390/molecules26133778)
Supplement: Supplementary file 1 [file molecules-26-03778-s001.zip › molecules-1242552-SI/molecules-1242552-supplementary-1.pdf]

*Supplementary Materials*

# Cost-effective production of L-DOPA by tyrosinase-immobilized polyhydroxyalkanoate nanogranules in engineered *Halomonas bluephagenesis* TD01

**Jiping Zhao [1], Ganqiao Ran [2], Mengmeng Xu [1], Xiaoyun Lu [1,*] and Dan Tan [1,*]**

[1] Department of Biological Science and Bioengineering, Key Laboratory of Biomedical Information Engineering of the Ministry of Education, School of Life Science and Technology, Xi'an Jiaotong University, Xi'an 710049, Shaanxi, People's Republic of China; zjp93823@sina.com (J.P.Z); yxmm11@stu.xjtu.edu.cn (M.M.X)

[2] Institute of Bio-Agriculture of Shaanxi Province, Xi'an 710043, Shaanxi, People's Republic of China; ranganqiao@ ms.xab.ac (G.Q.R)

* Correspondence: luxy05@mail.xjtu.edu.cn (X.Y.L); tandan@mail.xjtu.edu.cn (D.T)

**Table S1** The sgRNA sequences and primers used in this study

| Descriptions | Sequence (5'-3') |
|---|---|
| sgRNA sequence for *phaC*$_{Hb}$ knock out | ACTGCAAAAAGCCGACTGGC**TGG** |
| sgRNA sequence for *tyrVs-phaC*$_{Hb}$ fusion gene knock in | GGGAGAGTGAGTATGCTGTC**AGG** |
| **Primers for plasmid construction** | |
| sc-F | GGGGTGACGGCAATGTTATCACTAGTATTATACCTAGGACTGAGC |
| sc-R | GCAGTGGTGAAGTGGAAGGTATAAGCTTGCGGCCGCGTCGT |
| sg-F | GATAACATTGCCGTCACCCCGTTTTAGAGCTAGAAATAGC |
| sg-R | AAAGGCGTCCATCATTTTATCTTGCCGGGTTCAAAAAAAGCAC |
| C-up-F | CAAGATAAAATGATGGACGCCTTTAGT |
| C-up-R | TGACTCGCTGATCATTAGATGGTTATACTCACTCTCCCTCACCTGC |
| C-down-F | GCAGGTGAGGGAGAGTGAGTATAA |
| C-down-R | ACGACGCGGCCGCAAGCTTATACCTTCCACTTCACCACTGC |
| porin-Vs-F | GATCTGCCATCTAGTATTTCTCCTCTTTCTCTAGTAAAGTCTGCAGCTG |
| Vs-C-R | ACCCTGACAGGCTACCACCACCTGAACCACCACCT |
| phaC-Hb-F | TGGTGGTAGCCTGTCAGGGTGGAAAATGC |
| phaC-Hb-R | CTATAGGGCGAATTGGAGCTTTACGACGCGGGAAGCTC |
| vs-inf-F | TTATTACGACGCGGGAAGCTCACCTAA |
| vs-inf-R | ATGGCAGATCTCAATTGGGCGAAAT |
| vs-inv-F | TTAGGTGAGCTTCCCGCGTCGTAATAACCATCTAATGATCAGCGAGTCATG |
| vs-inv-R | ATTTCGCCCAATTGAGATCTGCCATACTCACTCTCCCTCACCTGCTTGGC |
| sgVs-F | GGGAGAGTGAGTATGCTGTCGTTTTAGAGCTAGAAATAGCAAGTT |
| sgVs-R | GACAGCATACTCACTCTCCCACTAGTATTATACCTAGGACTGAGC |
| **Primers for detection and mRNA analysis** | |
| C-test-F | ACTCCAGGCTCATGCCAAGG |
| C-test-R | ATCAGCGGTCATGGCTTCCA |
| 16sRNA-qF | ATCGGGAGGAATACCAGTG |
| 16sRNA-qR | CGTTTACGGCGTGGACTA |
| phaCHb-qF | CCGGTGACGTTTGTGTTG |
| phaCHb-qR | TTATGGGGTGGGTTCACAAT |
| Vs-qF | TATGCGGCTGGCTTTCTAA |
| Vs-qR | GCAAAACCGCGAACCATA |

The underlined bold base is PAM sequence

**Table S2** Result of mass spectrometer-based quantitative proteomics (see attached file)

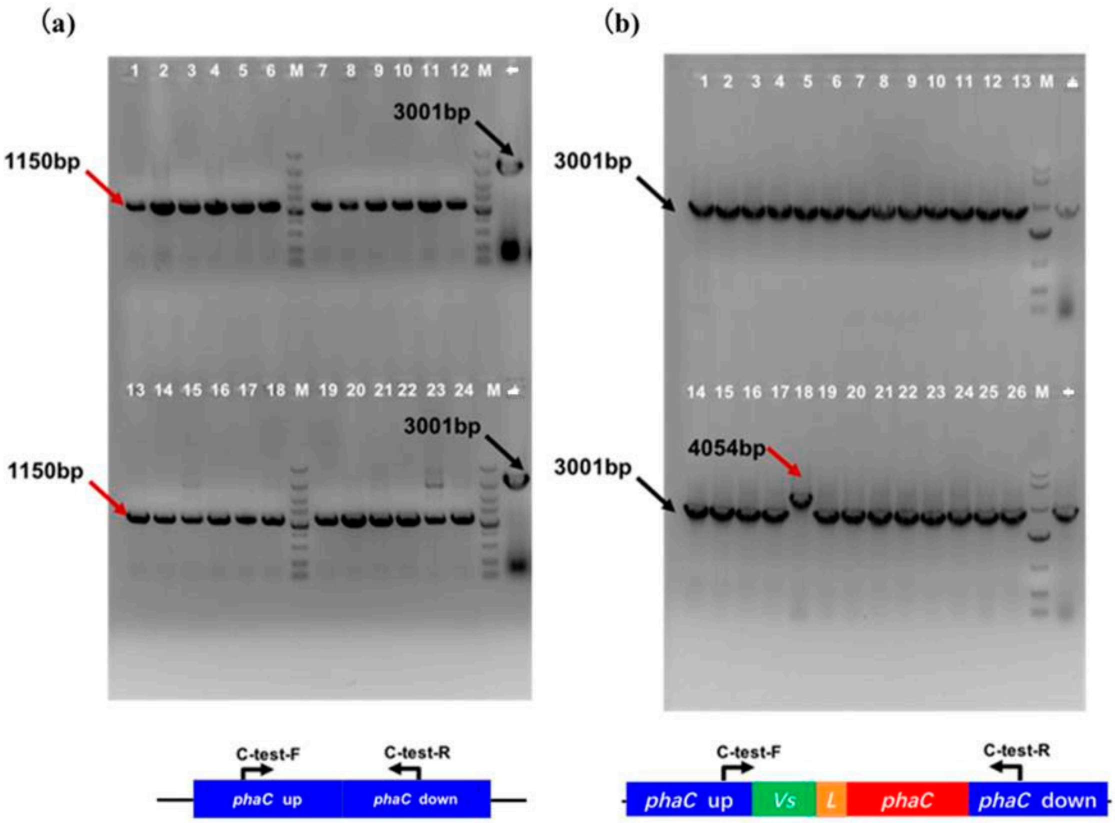


**Figure S1** The identification of $phaC_{Hb}$ knock-out (a) and $tyrVs\text{-}phaC_{Hb}$ fusion gene knock-in (b).

(a) Identification of $phaC_{Hb}$ knock-out mutants by colony PCR. All the tested clones showed a positive shorter band of 1150 bp when the detection primer C-test-F & C-test-R located outside of $phaC_{Hb}$ gene was used. A negative band (-) with a length of 3001 bp was obtained using *H. bluephagenesis* TD01 genome as the template.

(b) Identification of $tyrVs\text{-}phaC_{Hb}$ fusion gene knock-in mutants by colony PCR. One of the 26 tested clones showed a positive longer band of 4054 bp when the detection primer C-test-F & C-test-R was used. A negative band (-) with a length of 3001 bp was obtained using *H. bluephagenesis* TD01 genome as the template. M: DL10000 DNA ladder (Takara, Japan).

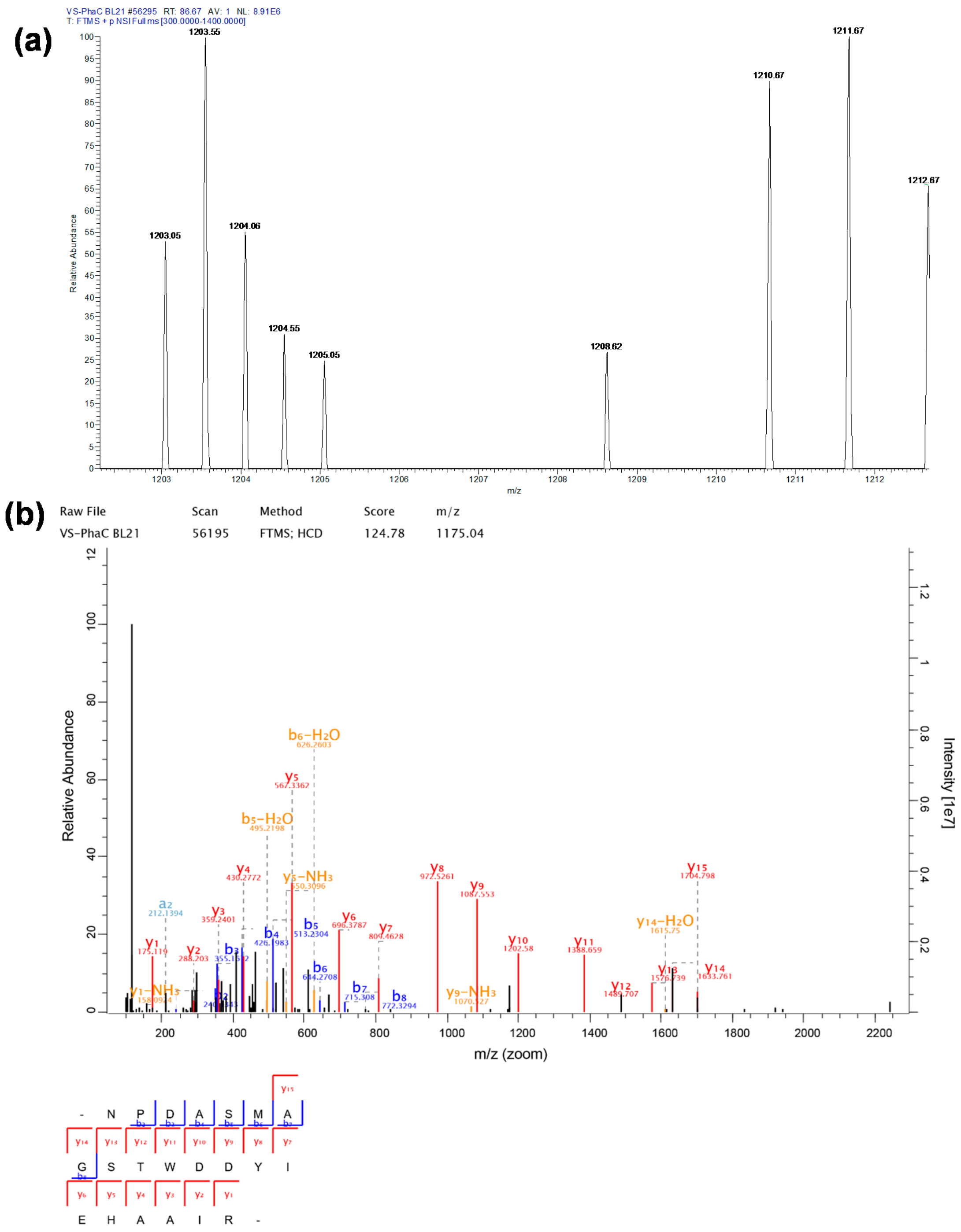


**Figure S2** Absolute quantification of fused TyrVs on the surface of PHA-TyrVs nanogranules produced by *E. coli*-P-Vs using mass spectrometer-based quantitative proteomics

(a) The MS 1 spectra of the peptide NPDASMAGSTWDDYIEHAAIR (m/z 1175.04) of TyrVs protein.

(b) The MS 2 spectra of the light precursor ion upon CID.

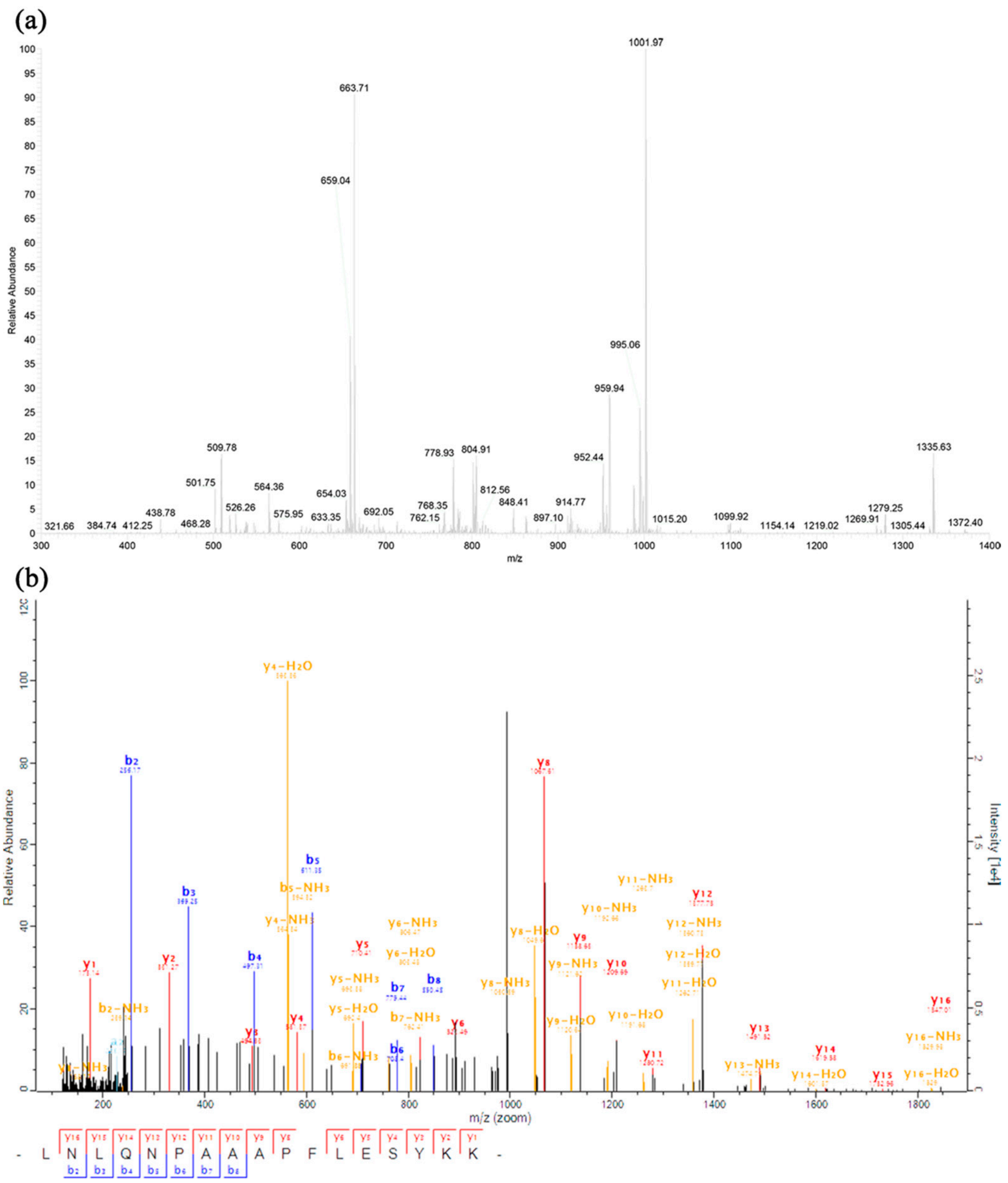


**Figure S3** Absolute quantification of fused TyrVs on the surface of PHA-TyrVs nanogranules produced by *H.b*-P-Vs using mass spectrometer-based quantitative proteomics

(a) The MS 1 spectra of the peptide LNLQNPAAAPFLESYKK (m/z 995.06) of TyrVs protein.

(b) The MS 2 spectra of the light precursor ion upon CID.

(a)

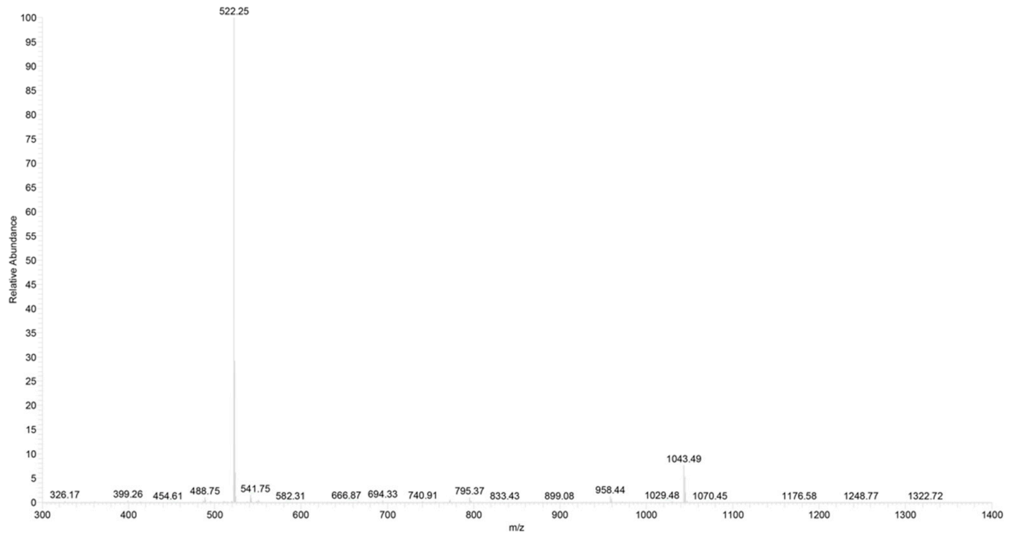


(b)

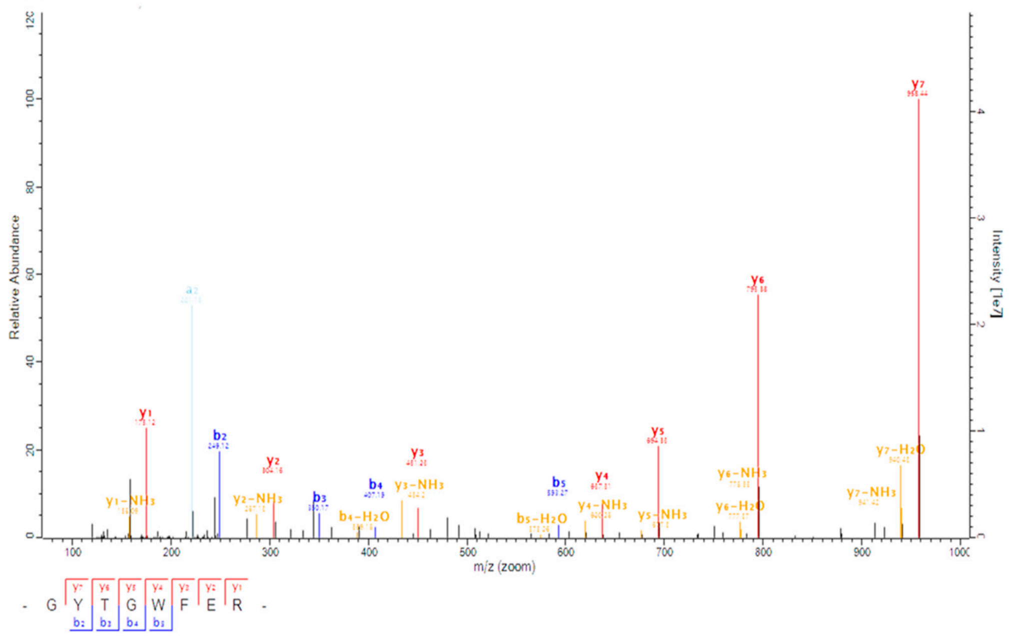


**Figure S4** Absolute quantification of fused TyrVs on the surface of PHA-TyrVs nanogranules produced by *H.b*-G-Vs using mass spectrometer-based quantitative proteomics.

(a) The MS 1 spectra of the peptide GYTGWFER (m/z 552.25) of TyrVs protein.

(b) The MS 2 spectra of the light precursor ion upon CID.
